# Recruitment to oral steroids for the resolution of otitis media with effusion in children (OSTRICH) study: challenges of a randomised controlled trial in secondary care sites across Wales and England

**DOI:** 10.1186/1745-6215-16-S2-P119

**Published:** 2015-11-16

**Authors:** Cherry-Ann Waldron, Emma Thomas-Jones, Debbie Harris, Vicky Shepherd, Rebecca Cannings-John, Kerry Hood, Nick Francis, Chris Butler

**Affiliations:** 1 South East Wales Trials Unit, Cardiff University, Cardiff, UK; 2 Institute of Primary Care and Public Health, Cardiff University, Cardiff, UK; 3 Nuffield Department of Primary Care Health Sciences, University of Oxford, Oxford, UK

## Introduction and aims

OSTRICH is a randomised controlled trial for otitis media with effusion (OME) in children, to determine the clinical and cost effectiveness of a short course of oral steroids. The aim is to describe and evaluate factors that have contributed to lower than expected recruitment rates.

## Method

Challenges faced to recruitment include the fluctuating and seasonal nature of OME, bringing together of two separate clinical services (Audiology and ENT), limited capacity for additional appointments for follow ups, and the efficiency of children’s hospitals in terms of grommet surgery waiting times.

## Results

Recruitment opened in March 2014 in Wales. However due to low recruitment rates, sites were extended into England. The trial now has 22 sites, 18 are actively recruiting, of those not recruiting are the children’s hospitals. As of 30th April 2015, 404 children have been screened, 255 (63%) have been eligible, with 206 (81%) recruited into the trial. The trial has a 96% successful follow up rate at 5 week timepoint and 89% at 6 months. Variation in recruitment rates across sites is a result of differences in how ENT and Audiology services are set up in secondary care and how the study is being incorporated into these services.

## Conclusion

Improvement in recruitment is due to opening more sites, continuous liaison with site staff and research nurses, and facilitating recruitment as much as possible by revisiting inclusion criteria and extending the time window for audiology confirmed hearing loss. Lessons learned here provide valuable insight into how recruitment can be optimised for future trials.

